# Effects of Fertilization and Sampling Time on Composition and Diversity of Entire and Active Bacterial Communities in German Grassland Soils

**DOI:** 10.1371/journal.pone.0145575

**Published:** 2015-12-22

**Authors:** Sarah Herzog, Franziska Wemheuer, Bernd Wemheuer, Rolf Daniel

**Affiliations:** 1 Department of Genomic and Applied Microbiology & Göttingen Genomics Laboratory, Institute of Microbiology and Genetics, Georg-August-University Göttingen, Grisebachstr. 8, 37077 Göttingen, Germany; 2 Section of Agricultural Entomology, Department for Crop Sciences, Georg-August-University of Göttingen, Grisebachstr. 6, 37077 Göttingen, Germany; University of Tartu, ESTONIA

## Abstract

Soil bacteria are major players in driving and regulating ecosystem processes. Thus, the identification of factors shaping the diversity and structure of these communities is crucial for understanding bacterial-mediated processes such as nutrient transformation and cycling. As most studies only target the entire soil bacterial community, the response of active community members to environmental changes is still poorly understood. The objective of this study was to investigate the effect of fertilizer application and sampling time on structure and diversity of potentially active (RNA-based) and the entire (DNA-based) bacterial communities in German grassland soils. Analysis of more than 2.3 million 16S rRNA transcripts and gene sequences derived from amplicon-based sequencing of 16S rRNA genes revealed that fertilizer application and sampling time significantly altered the diversity and composition of entire and active bacterial communities. Although the composition of both the entire and the active bacterial community was correlated with environmental factors such as pH or C/N ratio, the active community showed a higher sensitivity to environmental changes than the entire community. In addition, functional analyses were performed based on predictions derived from 16S rRNA data. Genes encoding the uptake of nitrate/nitrite, nitrification, and denitrification were significantly more abundant in fertilized plots compared to non-fertilized plots. Hence, this study provided novel insights into changes in dynamics and functions of soil bacterial communities as response to season and fertilizer application.

## Introduction

Soil bacteria play important roles in ecosystem functioning and processes such as biogeochemical cycles and nutrient transformation [1–3]. Moreover, they have a severe impact on plant productivity (as reviewed in [1, 4]). Thus, it is crucial to understand how these communities support the stability of ecosystem processes [5–7] and to identify factors shaping the diversity and structure of soil bacterial communities.

It is well-known that different soil properties such as pH or soil moisture influence bacterial communities in grassland soils [8–12]. In addition, different management regimes changed bacterial community composition and diversity in grassland soils [12–15]. Fierer et al. [13] investigated soil microbial communities across nitrogen gradients by pyrotag sequencing. Nitrogen amendment did not affect the soil bacterial diversity but significantly altered the community composition. In contrast, Nacke et al. [12] observed the highest diversity of soil bacteria in fertilized intensively managed grasslands. However, the majority of these studies used DNA-based approaches. Thus, they focused on the entire bacterial community, which also contains dead cells, extracellular DNA, and dormant microorganisms [16]. Correspondingly, still little is known on the potentially metabolic active bacterial communities in grassland soils and their response to changing environmental conditions. These communities can be assessed by analysis of 16S rRNA transcripts (e.g. [17]). It should be noted that rRNA abundance serves as an index for activity but not as a direct measure of activity (reviewed in [18]). Thus, changes in the relative abundance do not necessarily reflect changes in the activity of the studied organisms.

Previous studies showed that the structure of bacterial communities in grassland soils is altered by sampling time and season [14, 19–21]. The bacterial community structure in an upland grassland soil analyzed by automated ribosomal intergenic spacer analysis (ARISA) was influenced by season [14]. This result was supported by a study of Habekost et al. [21], who observed distinct seasonal variations in microbial community structure of a temperate grassland soil. The authors suggest that these changes are driven by the availability and quality of organic resources. The analysis of soil microbial communities across different land-use types revealed that temporal shifts in community composition were often correlated with temperature conditions, which directly or indirectly regulate the structure of soil bacterial communities [11].

The aim of this study was to investigate the influence of fertilizer application and sampling time on entire (DNA-based) and potentially active (RNA-based) bacterial communities in German grassland soil. Therefore, soil samples were taken in April, July, and September over two consecutive years (2010 and 2011). We applied large-scale amplicon sequencing-based analysis of the V2-V3 region of the 16S rRNA genes and gene transcripts to assess the diversity and structure of entire and active bacterial communities. The combination of DNA-based and RNA-based analyses with next-generation-sequencing is unique in the study of bacterial community structure and diversity in grassland soils. We hypothesized that the entire and active communities are differently influenced by fertilizer application (hypothesis I). We further hypothesized that bacterial diversity remained consistent throughout the year, whereas the structure is shaped by season (hypothesis II). Moreover, we used this unique dataset to perform functional predictions with Tax4Fun and examined soil microbial functions and metabolic capabilities of the entire and the active bacterial communities. We hypothesized that fertilizer application changes the community structure and this is accompanied by changes in bacterial functions (hypothesis III).

## Material and Methods

### Study site

This study was carried out within the GrassMan experiment in the Solling Uplands, Germany (51°44´ N, 9°32´´E, 490 m a.s.l.). It was established in June 2008 [22]. The GrassMan experimental field belonged to the institution (Georg-August-University Göttingen). Therefore, no special permit was required for soil sampling. Endangered species were not affected by the sampling. The design of the GrassMan experiment included three levels of sward compositions (species-rich, monocot-reduced, and dicot-reduced), two mowing frequencies (once vs. three times per year), and two fertilizer treatments (fertilized with NPK vs. non-fertilized as reference). The experimental setup is further described by Petersen et al. [22]. The soil of the experimental area is a stony Haplic Cambisol [23]. During the study period, mean annual temperature and annual precipitation were 6.6°C and 732 mm in 2010 and 8.91°C and 724 mm in 2011, respectively (S1 Table).

### Sampling and soil characterization

Soil samples were collected from three fertilized (fe) and three non-fertilized (nf), species-rich plots mown once a year. Three soil cores (8 cm in diameter, depth 20 cm) per plot were taken and pooled. To analyze the effect of sampling time, samples were collected in spring (April; Apr), summer (July; Jul), and autumn (September; Sep) 2010 (10) and 2011 (11). Soil samples were frozen in liquid nitrogen and stored at -80°C until analysis. For determination of soil properties, subsamples from the pooled soil samples were dried at 60°C for seven days and sieved to < 2mm. Soil organic carbon (C) and total nitrogen (N) concentrations were determined from dried soil with a CN elemental analyzer (Elemental Analyzer EA 1108, Carlo Erba Instruments, Rodano, Italy). The gravimetric soil water content (%) was calculated from oven-dried subsamples. Soil pH values were measured from a soil water suspension ratio of 1:2 (water contains 0.1 M KCl).

### Extraction of nucleic acids from soil and reverse transcription

Total environmental RNA and DNA were co-extracted from 0.5 g soil per sample employing the RNA PowerSoil total RNA isolation kit and the RNA PowerSoil DNA elution accessory kit, respectively, as recommended by the manufacturer (MoBio Laboratories, Carlsbad, CA, USA). For RNA purification, residual DNA was removed with the TURBO DNA-free™ kit (Ambion Applied Biosystems, Darmstadt, Germany) from the extracted RNA. The absence of DNA was confirmed by PCR as described by Wemheuer et al. [24]. The DNA-free RNA was purified and concentrated using the RNeasy MinElute cleanup kit (Qiagen GmbH, Hilden, Germany). Isolated DNA was purified with the PowerClean DNA cleanup kit (MoBio Laboratories). DNA and RNA concentrations were determined using a NanoDrop ND-1000 spectrophotometer (Peqlab Biotechnologie GmbH, Erlangen, Germany). Approximately 500 ng of purified RNA was converted to cDNA using the SuperScript^TM^ III reverse transcriptase as recommended by the supplier (Invitrogen, Karlsruhe, Germany) and the reverse primer V3rev [25] of the subsequent PCR reaction.

### Amplification and sequencing of 16S rRNA gene regions and sequencing

The V2-V3 region of the 16S rRNA gene was amplified by PCR. The PCR reaction mixture (25 μl) contained 5-fold Phusion GC buffer, 200 μM of each of the four deoxynucleoside triphosphates, 5% DMSO, 0.4 μM of each primer, 0.5 U of Phusion Hot Start HF DNA polymerase (Fisher Scientific GmbH, Schwerte, Germany), and 25 ng of isolated DNA or cDNA as template. The V2-V3 region was amplified with the following set of primers modified by Schmalenberger [25] containing the Roche 454-pyrosequencing adaptors, key sequences and one unique MID (underlined) per sample: V2for 5’-CGTATCGCCTCCCTCGCGCCATCAG-(dN)_10_-AGTGGCGGACGGGTGAGTAA-3’ and V3rev 5’-CTATGCGCCTTGCCAGCCCGCTCAG-(dN)_10_-CGTATTACCGCGGCTGCTGG-3’. The following cycling conditions were used for the amplification of cDNA: initial denaturation at 98°C for 5 min and 25 cycles of denaturation at 98°C for 10 s, annealing at 72°C for 10 s and extension at 72°C for 10 s, followed by a final extension at 72°C for 5 min. For DNA amplification, the following cycling scheme was used: initial denaturation at 98°C for 5 min and 25 cycles of denaturation at 98°C for 45 s, annealing at 72°C for 30 s and extension at 72°C for 30 s, followed by a final extension at 72°C for 10 min. PCR reactions were performed in triplicate for each sample. The resulting PCR products were purified using the peqGold gel extraction kit (Peqlab Biotechnologie GmbH, Erlangen, Germany) and pooled in equal amounts. Obtained PCR products were quantified using the Quant-iT dsDNA HS assay kit and a Qubit fluorometer (Invitrogen GmbH) as recommended by the manufacturer. The Göttingen Genomics Laboratory determined the sequences of the partial 16S rRNA genes employing the Roche GS-FLX 454 pyrosequencer with Titanium chemistry as recommended by the manufacturer (Roche, Mannheim, Germany).

### Processing of 16S rRNA sequence data

Pyrosequencing-derived 16S rRNA gene (DNA) and transcript (RNA) datasets were processed and analyzed using the QIIME software package version 1.6 [26]. Sequences shorter than 200 bp, low quality sequences, and sequences with homopolymers (> 8 bp) were removed from the datasets. Pyrosequencing noise was removed using Acacia 1.52 [27]. Primer sequence residues were truncated using cutadapt version 1.0 [28]. Chimeric sequences were detected and eliminated using UCHIME 7.0.190 in *de novo* and in reference mode with the Silva SSURef 119 NR database as reference database [29, 30]. All remaining sequences were subsequently clustered in operational taxonomic units (OTUs) at 3% (species level) and 20% genetic distance (phylum level) using the QIIME pick_otus.py script and uclust [26]. OTUs represented by only a single sequence in the entire dataset (singletons) were removed (see [31]). Taxonomic assignment was performed via BLAST alignment against the most recent SILVA database (SSURef NR 119) [30]. Rarefaction curves, alpha diversity indices (Chao1, Shannon, Simpson, and Michaelis-Menten-Fit), and beta diversity (Principle Component analyses) were determined using QIIME according to Wemheuer et al. [32]. Functional profiles for each sample were predicted from 16S rRNA marker genes and gene transcripts in R (version 3.2.0; R Development Core Team 2015 [http://www.R-project.org/]) [33] using Tax4Fun with short read mode disabled [34].

### Statistical analysis

T-test for normal distributed data or the Mann-Whitney-test for not normal distributed data were performed using SigmaPlot version 11.0 (Systat Software GmbH, Erkrath, Germany). To compare taxonomic groups with soil properties, Spearman’s rank correlation coefficient was determined in SigmaPlot version 11.0. All other statistical analyses were conducted employing R version 3.2.0 [33]. Effects of fertilizer application on environmental properties and bacterial community were tested as described by Wemheuer et al [32]. In brief, the Student's two-sample *t*-test (homogenous variances) or the Welsh's two-sample *t*-test (heterogeneous variances) were employed for normally distributed samples. For non-normally distributed samples, the Wilcoxon–Mann–Whitney test was used. Changes in community structure and significant differences between samples and treatments were examined employing the metaMDS and envfit functions within the vegan package [35] as described by Wietz et al. [36]. Changes in community functions were studied by principal component analysis using the rda function within the same R package. Entire and active bacterial communities were analyzed separately as DNA and RNA were extracted from the same soil samples and thus represent spatial pseudo-replicates. The results of the statistical tests were regarded as significant at P values ≤ 0.05.

### Sequence data deposition

Sequence data were deposited in the Sequence Read Archive (SRA) of the National Center for Biotechnology Information (NCBI) under the accession number SRP041803.

## Results and Discussion

### Soil properties

In this study, the influence of season and fertilizer application on bacterial communities was assessed over two consecutive years. Water content in investigated soil samples varied between 12.6 and 34.0% (S2 Table). In 2010, water content was twofold higher in April and September than in July due to higher temperatures and dryer conditions during summer (S1 Table). The soil pH values ranged from 4.6 to 4.9. Statistical analysis revealed no significant differences of pH values between fertilized and non-fertilized plots. The C/N ratios varied between 11.1 and 15.2, which is typical for field conditions with a soil texture of loamy silt.

### General characteristics of the 16S rRNA datasets

To analyze and compare active and entire bacterial community structure and diversity, DNA and RNA were isolated from a total of 72 soil samples. Bacterial community composition and diversity were assessed by amplicon-based analyses of the V2-V3 region of the 16S rRNA gene and the corresponding transcript. After quality filtering, denoising, and removal of potential chimeras and non-bacterial sequences, 2,386,234 high-quality sequences with an average read length of 359 bp were obtained and used for further analyses (S3 Table). All sequences were classified below phylum level. The number of sequences per sample ranged from 11,804 to 72,754 (DNA level) and from 17,919 to 72,380 (RNA level). To perform analysis at equal surveying effort 11,800 sequences per sample were randomly selected and subsequently clustered into operational taxonomic units (OTUs) at species (3% genetic distance) and phylum level (20% genetic distance) (S1 and S2 Figs). Calculated rarefaction curves at DNA and RNA level reached saturation at phylum level indicating that the major part of the bacterial diversity was recovered by the surveying effort (S1 Fig). This is supported by the calculated coverage of approximately 80% at phylum level (S4 and S5 Tables). At species level, approximately 43% of the bacterial richness was recovered (S6 and S7 Tables).

### Composition of active and entire bacterial community

**Fig 1:**
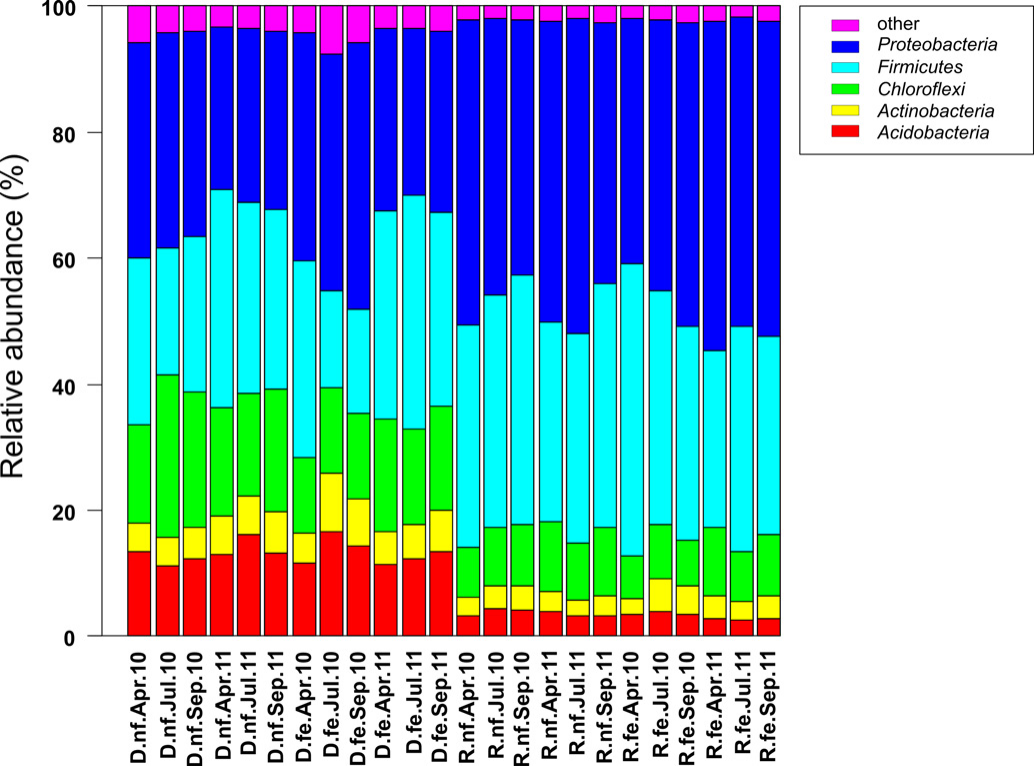
Relative abundances of bacterial phyla (> 1%) derived from the analyzed soil samples. Phyla accounting for less than 1% of all sequences are summarized in the group “other”. Fertilized (fe) and non-fertilized (nf) samples are shown. Samples were taken in April (Apr), July (Jul), and September (Sep) in 2010 (10) and 2011 (11). The entire (D) and active (R) bacterial communities were analyzed.

Obtained sequences were assigned to 41 bacterial phyla, 150 classes, and 374 families (Fig 1). Five dominant phyla (> 1% abundance) were present in each soil sample and accounted for more than 96% of all bacterial sequences analyzed in this study. Rare phyla are shown in S3 Fig. *Proteobacteria* were predominant across all samples (DNA 31.2%, RNA 45.3%). The active bacterial community was dominated by *Alphaproteobacteria* (37.2%) and *Firmicutes* (36.0%) whereas the entire bacterial community was dominated by *Firmicutes* (27.4%), *Alphaproteobacteria* (15.9%), *Chloroflexi* (17%), *Acidobacteria* (13.3%), and *Actinobacteria* (6.0%). These results are in agreement with previous studies on bacterial community composition in grassland soils [11, 12, 37]. However, studies investigating the active bacterial community in combination with next-generation-sequencing technologies in grassland soils are rare as most previous researchers used DNA-based approaches only (e.g. [12, 13, 37]).

**Fig 2:**
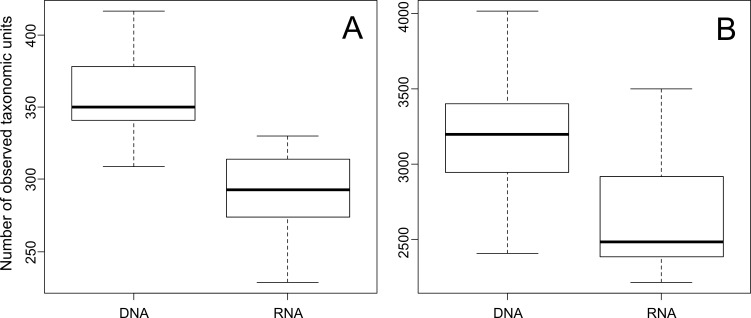
Number of observed taxonomic units in the entire and active bacterial community. **A**. estimated OTUs at phylum level and **B**. estimated OTUs at species level. Depicted are OTUs estimated for the entire (D) and active (R) bacterial community.

We found significant differences between the number of OTUs derived from 16S rRNA genes and 16S rRNA gene transcripts (Fig 2). At phylum and species level, the number of OTUs at DNA level (358 and 3,159 OTUs, respectively) was significantly higher (p <0.001) compared to RNA level (292 and 2,674 OTUs, respectively). In conclusion, the active community was less diverse than the entire community. This is consistent with the results of Baldrian et al. [17], who investigated the active and the entire bacterial community in forest soils. They found a stronger dominance of fewer phyla in the RNA dataset compared to the DNA-derived dataset. They encountered 1,500 (DNA) and 1,200 OTUs (RNA) at species level. This is also in accordance with a study on prokaryotic communities in dryland soils [38].

**Fig 3:**
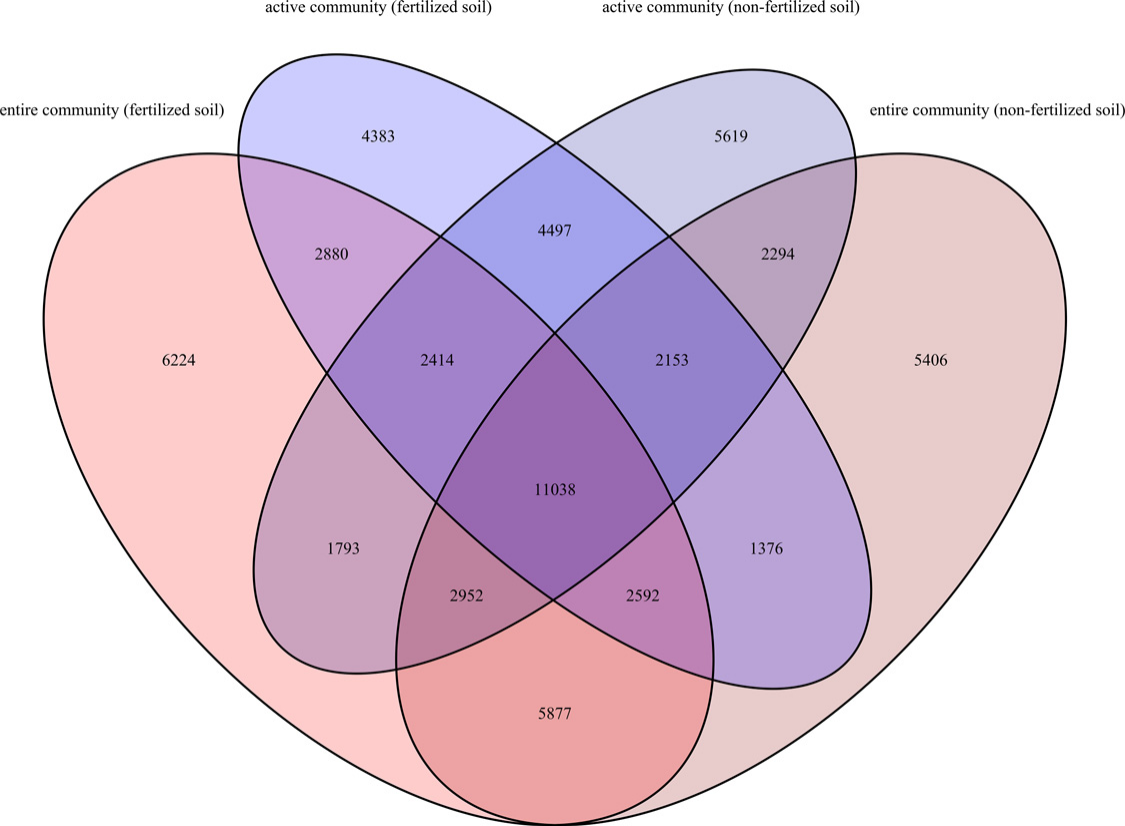
Venn diagram of all analyzed OTUs in fertilized and non-fertilized soils at entire and active bacterial community level. Depicted were OTUs estimated at entire community level (fertilized soil), active community level (fertilized soil), entire community level (non-fertilized soil), and active community level (non-fertilized soil) and all other possible interfaces.

Analysis of bacterial community composition revealed that 11,038 OTUs at species level were shared between the entire and active bacterial community in fertilizer and non-fertilizer treatments. This core community comprised approximately 90% of all analyzed sequences (Fig 3). More than 21,632 OTUs were unique (present at DNA or RNA level or in fertilized or non-fertilized plots) and accounted for only 1% of all analyzed sequences.

The most abundant OTU at species level in the active and entire bacterial community belonged to the genus *Bacillus* (phylum *Firmicutes*), which comprised 15.3% (RNA level) and 12.5% (DNA level) of all analyzed sequences. Members of *Bacillus* are most common in grassland soils and well adapted to this environment [39, 40]. Representatives of the *Bacillus* genus improve plant health due to their ability to produce substances that suppress pests and pathogens [41]. At RNA level, the second most abundant OTU (12.5%) was classified as member of the *Acetobacteraceae* (*Proteobacteria*). This family is recognized by its ability to oxidize ethanol to acidic acid in acidic and neutral media [42]. As members of this family can use a wide range of substrates such as glucose, ethanol, lactate or glycerol as energy source, they are important microorganisms in food industry such as the vinegar production [43]. Furthermore, members of this family exhibit optimal growth conditions at low pH values [44] as observed in our study.

### Correlations between abundant bacterial groups and soil properties in fertilized and non-fertilized soils

**Fig 4:**
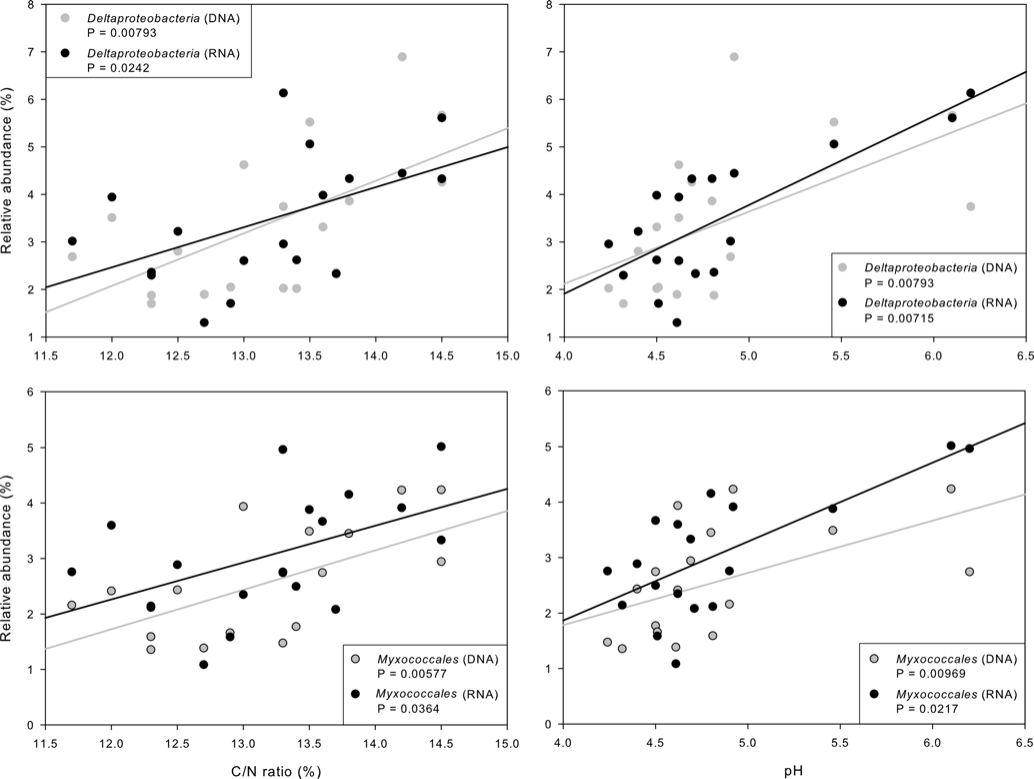
Spearman´s rank correlations between relative abundances of the class *Deltaproteobacteria* and the order *Myxococcales* derived from DNA and RNA dataset with pH and C/N ratio in the fertilizer treatment. Regression lines were included for RNA in black and DNA in gray. P values are shown for the active (RNA) and entire (DNA) *Deltaproteobacteria* **and *Myxococcales***.

We used Spearman’s rank correlation to analyze the relationship between soil properties and relative abundances of dominant phyla, proteobacterial classes, and orders (Tables 1–4). We tested all of these phylogenetic groups with more than 1% abundance in the complete dataset. Several phyla and proteobacterial classes correlated with environmental properties (Tables 1 and 2). In the fertilized plots, the active part of the *Chloroflexi* correlated significantly positive with pH and C/N. In addition, *Firmicutes* showed a significant negative correlation with the C/N ratio. The opposite was observed for the *Alphaproteobacteria*. The *Deltaproteobacteria* correlated significantly positively with pH and C/N at active and entire community level (Fig 4). In the non-fertilized plots, the *Gammaproteobacteria* correlated significantly negative with the water content at entire community level, while *Deltaproteobacteria* correlated significantly positively with pH at active community level. Overall, the number of significant correlations was two-fold higher at RNA level than at DNA level, This indicated that the active bacterial community is more sensitive to changes in soil properties than the entire community. Moreover, the bacterial community is stronger influenced by soil properties in fertilized compared to non-fertilized soils.

**Table 1:** Spearman´s Rank correlations of the abundance of the most abundant phyla, proteobacterial classes and soil properties in fertilized soils. Relative abundances derived from the active (RNA) and entire (DNA) bacterial community were separately analyzed. Bold numbers indicate P values < 0.05.

Group	Correlation					
	pH		Water content		C/N	
	DNA	RNA	DNA	RNA	DNA	RNA
*Acidobacteria*	0.302	0.347	-0.188	-0.155	0.170	0.375
*Actinobacteria*	0.139	0.394	-0.373	-0.413	0.258	0.418
*Chloroflexi*	0.085	**0.480**	0.123	-0.131	-0.148	**0.489**
*Firmicutes*	-0.299	-0.333	0.298	0.149	-0.260	**-0.621**
*Alphaproteobacteria*	-0.363	0.013	-0.226	0.023	-0.086	**0.481**
*Betaproteobacteria*	0.244	0.246	-0.319	-0.079	0.326	0.407
*Gammaproteobacteria*	-0.001	0.149	0.004	0.045	0.137	0.125
*Deltaproteobacteria*	**0.604**	**0.611**	0.039	0.010	**0.604**	**0.528**

**Table 2:** Spearman´s Rank correlations of the abundance of the most abundant phyla, proteobacterial classes and soil properties in non-fertilized soils. Relative abundances derived from the active (RNA) and entire (DNA) bacterial community were separately analyzed. Bold numbers indicate P values < 0.05.

Group	Correlation					
	pH		Water content		C/N	
	DNA	RNA	DNA	RNA	DNA	RNA
*Acidobacteria*	-0.323	-0.176	0.102	0.110	0.238	0.388
*Actinobacteria*	-0.096	-0.043	-0.158	-0.110	-0.033	0.121
*Chloroflexi*	-0.437	0.076	-0.309	-0.238	-0.309	0.377
*Firmicutes*	0.020	-0.298	0.156	0.323	0.003	-0.322
*Alphaproteobacteria*	-0.187	0.083	0.088	-0.282	0.166	0.304
*Betaproteobacteria*	0.347	0.390	-0.247	-0.117	-0.205	0.095
*Gammaproteobacteria*	0.344	0.373	**-0.515**	0.273	-0.047	-0.009
*Deltaproteobacteria*	0.279	**0.544**	0.102	0.158	-0.437	-0.002

**Table 3:** Spearman´s Rank correlations of the abundance of the most abundant orders and soil properties in fertilized soils. Relative abundances derived from the active (RNA) and entire (DNA) bacterial community were separately analyzed. Bold numbers indicate P values < 0.05.

Group	Correlation					
	pH		Water content		C/N	
	DNA	RNA	DNA	RNA	DNA	RNA
*Acidobacteriales*	-0.342	**-0.568**	0.177	0.151	-0.09	-0.291
Subgroup 3	-0.004	0.206	0.034	-0.201	-0.141	0.374
Subgroup 7	0.278	**0.618**	-0.053	-0.163	0.013	0.410
*Frankiales*	0.039	0.290	-0.313	-0.418	0.051	**0.487**
S085_uncultured bacterium	0.361	0.286	-0.305	0.040	0.330	**0.644**
*Ktedonobacterales*	-0.316	0.321	0.219	-0.332	-0.391	**0.624**
AG30-KF-AS9	-0.358	-0.444	0.104	-0.136	-0.457	-0.424
JG37_AG-4_uncultered bacterium	0.222	0.087	0.125	0.236	-0.061	0.100
D4-96_unculutred bacterium	**0.523**	**0.539**	-0.258	-0.260	0.319	0.453
*Bacillales*	-0.285	-0.339	0.305	0.171	-0.332	**-0.645**
*Clostridiales*	-0.197	-0.227	-0.158	-0.255	0.302	-0.036
*Myxococcales*	**0.591**	**0.536**	0.052	0.012	**0.623**	**0.494**
*Burkholderiales*	0.129	0.173	-0.132	0.056	**0.479**	0.217
*Caulobacterales*	-0.221	-0.336	-0.201	-0.034	0.116	0.259
*Rhizobiales*	0.377	0.041	-0.180	-0.336	0.349	0.186
*Rhodospirillales*	-0.457	0.061	-0.146	0.035	-0.277	**0.500**
*Xanthomonadales*	0.120	0.147	-0.177	-0.033	-0.085	0.130

**Table 4:** Spearman´s Rank correlations of the abundance of the most abundant orders and soil properties in non-fertilized soils. Relative abundances derived from the active (RNA) and entire (DNA) bacterial community were separately analyzed. Bold numbers indicate P values < 0.05.

Group	Correlation					
	pH		Water content		C/N	
	DNA	RNA	DNA	RNA	DNA	RNA
*Acidobacteriales*	**-0.469**	-0.347	0.075	0.0114	0.171	0.378
Subgroup 3	-0.380	-0.189	0.077	-0.075	0.159	0.308
Subgroup 7	0.131	0.067	-0.009	-0.209	0.236	0.084
*Frankiales*	-0.193	-0.244	0.146	-0.307	0.008	0.086
S085_uncultured bacterium	0.345	0.388	-0.410	0.0568	-**0.503**	-0.137
*Ktedonobacterales*	-0.231	-0.004	0.009	-0.366	-0.009	0.194
AG30-KF-AS9	-0.253	-0.193	-0.006	-0.197	-0.248	0.024
JG37_AG-4_uncultered bacterium	-0.002	-0.285	-0.383	-0.110	-0.180	**0.491**
D4-96_unculutred bacterium	0.0228	**0.534**	-0.284	-0.309	-0.453	-0.110
*Bacillales*	-0.045	-0.399	0.214	0.187	0.023	-0.349
*Clostridiales*	**0.685**	**0.480**	0.012	0.391	-0.130	-0.01
*Myxococcales*	0.253	**0.558**	0.133	0.162	-0.443	-0.013
*Burkholderiales*	0.227	0.093	-0.172	0.100	-0.042	0.164
*Caulobacterales*	0.095	0.115	-0.226	0.216	-0.117	0.448
*Rhizobiales*	-0.182	-0.135	0.168	0.096	0.217	0.374
*Rhodospirillales*	-0.215	0.064	0.100	-0.430	0.093	0.170
*Xanthomonadales*	0.275	0.375	-0.104	0.057	-0.129	-0.144

The most abundant orders of the active bacterial community in the fertilizer-treated soils were strongly correlated with soil properties (Tables 3 and 4). Active community members of the order *Acidobacteriales* (subgroup 1) were significant negatively correlated with pH. This is consistent with the results of a DNA-based study [45]. *Myxococcales* (*Deltaproteobacteria*) were significant positively correlated with pH and C/N (Fig 4). This is in line with a study of myxobacterial communities in different soils by Zhou et al. [46]. The authors observed a strong correlation between pH and the relative abundance of *Myxobacteria*. A key role in the soil carbon turnover is suggested for this phylogenetic group [47].

### Fertilizer application changed structure and diversity of the bacterial community

**Fig 5:**
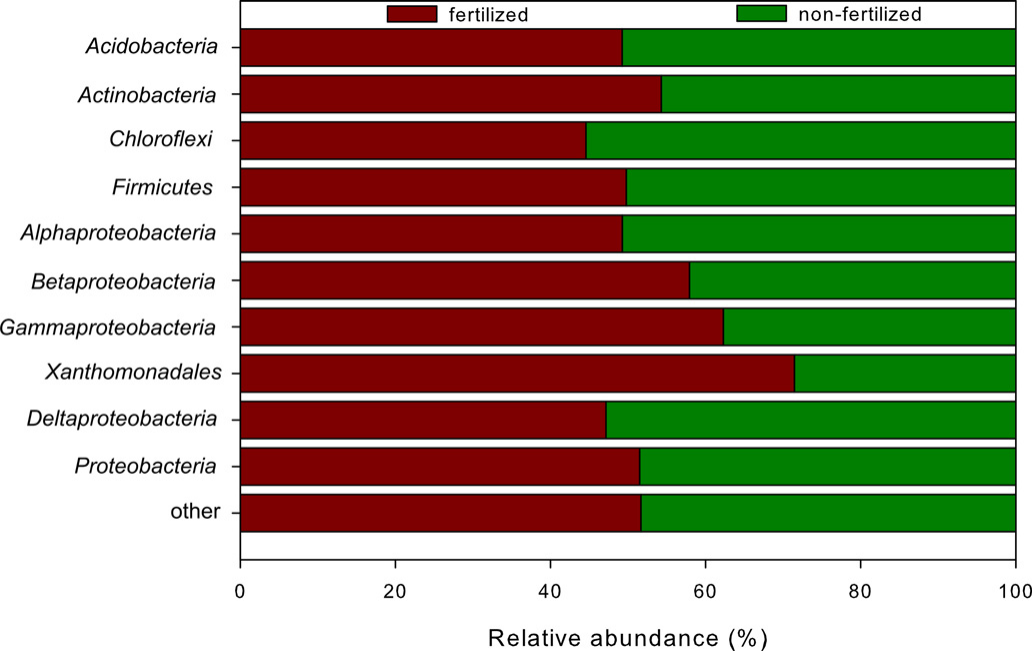
Relative abundances of bacterial phyla, proteobacterial classes, and *Xanthomonadales* derived from the fertilizer (red) and non-fertilizer (green) treatment. Depicted were phyla with more than 1% abundance. All other rare phyla were summarized in the group “other”.

To analyze the influence of fertilizer amendment on the bacterial community structure, we collected and analyzed samples from non-fertilized and fertilized plots over two consecutive years. We observed a higher number of *Actinobacteria*, *Betaproteobacteria*, and *Gammaproteobacteria* in the fertilized soils whereas *Acidobacteria*, *Chloroflexi*, *Firmicutes*, *Alphaproteobacteria*, and *Deltaproteobacteria* were more abundant in the non-fertilized plots (Fig 5). Within the *Gammaproteobacteria*, the order *Xanthomonadales* was significantly more abundant in the fertilized plots at entire and active community level. These organisms could also be beneficial for plant growth by increasing sulfur availability via oxidation and phosphate via solubilization [48]. This is consistent with previous studies investigating the impact of nitrogen fertilization on soil bacterial communities. *Gammaproteobacteria* increased with rising N inputs [49–51] or with long-term fertilization [52].

At entire bacterial community level, we observed higher numbers of OTUs in fertilized soils than in non-fertilized plots (3,265 and 3,053 OTUs, respectively). These results are in accordance with previous studies [12, 53, 54]. Nacke et al. [12] found similar OTU values in fertilized and non-fertilized grasslands. In contrast to this, we observed higher numbers of OTUs at the active bacterial community level in non-fertilized plots. The active bacterial community showed an opposite behavior in non-fertilized plots with 5% more OTUs at species level.

Until now, very little is known about the RNA-based analysis of the active bacterial community composition in grassland soils using next-generation-sequencing-technologies [17, 55]. Baldrian et al. [17] described differences between the active and entire bacterial community in forest soils and Pfeiffer et al. [55] investigated the active and entire bacterial community in a soil mesocosm experiment using beech and ash with and without litter overlay. Our study showed that fertilizer amendment impacts the active bacterial community and led to a diversity loss. This might be explained by increased activity of some groups, which can use N compounds for respiratory processes. It is of great importance to stabilze soil pH in fertilized soils for maintaining nutrient cycles [56]. In our study, fertilizer application was combined with phosphorus, potassium oxide, and calcium oxide (lime), which lead to stable soil pH values. In contrast, Kennedy et al. [57] investigated the impact of lime and nitrogen amendment on bacterial community structure in a microcosm experiment. They observed that the combined amendment of lime and nitrogen increased microbial activity whereas nitrogen amendment alone lead to a significant decrease of microbial activity in treated soils compared to non-treated soils. However, it is difficult to compare our data with the results of recent studies due to the fact that the number of analyzed sequences impacts the estimated number of OTUs [58]. In most previous studies, fewer sequences and other regions of the 16S rRNA gene have been analyzed [15, 53, 59] or different methods were applied [57].

### Sampling time influenced soil bacterial communities in different ways

**Fig 6:**
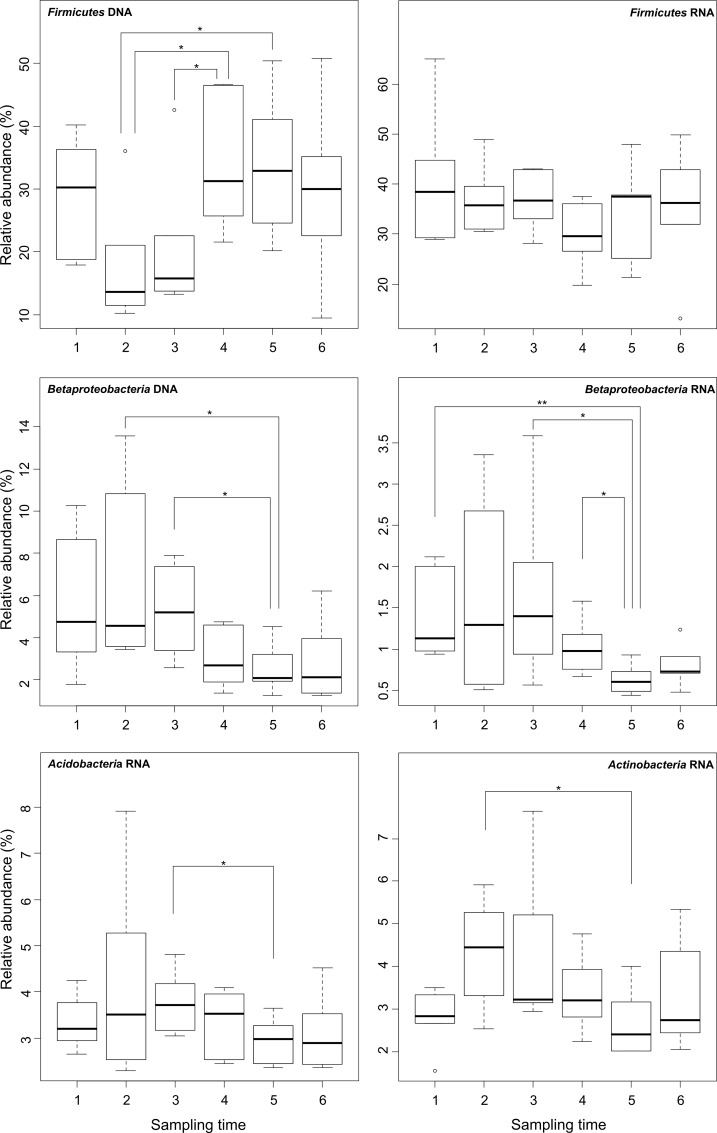
Boxplot diagram showing relative abundances of main phyla and proteobacterial classes over sampling time. **1**; April 2010, **2**; July 2010, **3**; September 2010, **4**; April 2011, **5**; July 2011, and **6**; September 2011. Asterisks indicate significant differences between sampling times in the active (RNA) and entire (DNA) bacterial phyla and proteobacterial classes.

To analyze the effect of sampling time on soil bacterial community structure, soil samples were collected in spring (April), summer (July), and autumn (September) over two consecutive years (2010 and 2011). *Acidobacteria*, *Actinobacteria*, *Firmicutes*, and *Betaproteobacteria* showed significant different abundances with respect to sampling time (Fig 6). *Firmicutes* showed only significant differences at entire bacterial community level, while *Betaproteobacteria* showed significant differences at active and entire community level. For *Actinobacteria* and *Acidobacteria*, a seasonal effect was determined at active community level but not at entire community level. *Chloroflexi*, *Alphaproteobacteria*, *Gammaproteobacteria* and *Deltaproteobacteria* were not affected by sampling time, which indicates that these groups are more recalcitrant to environmental changes.

**Fig 7:**
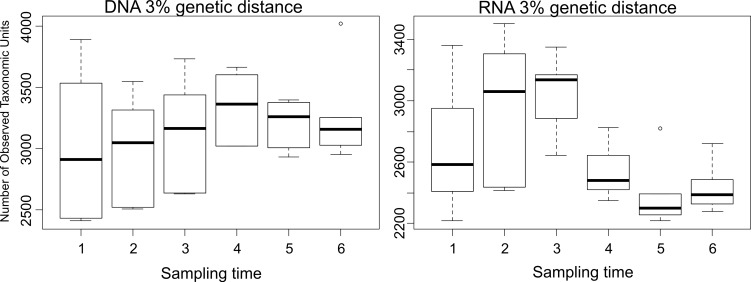
Boxplot diagram of the number of taxonomic units (OTUs) at species level over sampling time at DNA and RNA level. **1**; April 2010, **2**; July 2010, **3**; September 2010, **4**; April 2011, **5**; July 2011, and **6**; September 2011.

Moreover, we observed significant differences of OTU numbers at species level within the different sampling times at active bacterial community level but not at entire community level (Fig 7). Especially, at active community level, the summer samples in 2010 were significant different from the summer samples in 2011. A similar result was observed at phylum level (S4 Fig). These differences might be explained by seasonal changes of temperature, water availability, and plant growth activity. This is in line with the results of previous studies [14, 21, 60, 61]. A rainfall manipulating experiment showed little differences in soil bacterial community composition in grasslands after 5 years of manipulation [60]. Nevertheless, changes in microbial abundance and composition in response to extreme weather conditions were recorded. Interstingly, repeated sampling across seasons and years showed that these changes were only transient. Smit et al. [61] analyzed samples of an agricultural soil taken in all seasons and determined the bacterial community composition by cultivation and denaturing gradient gel electrophoresis (DGGE). The authors showed that the bacterial community in summer (July) differs from that in other seasons. They concluded that a stable microbial community existed although parameters such as humidity and nutrient supply shaped the bacterial communities. Our study showed minor differences of the entire bacterial community diversity and structure between the sampling times, but significant differences at active bacterial community level. We concluded that the response of changing environmental conditions were more pronounced and earlier visible at active than at entire bacterial community level.

### Functional Analysis

**Fig 8:**
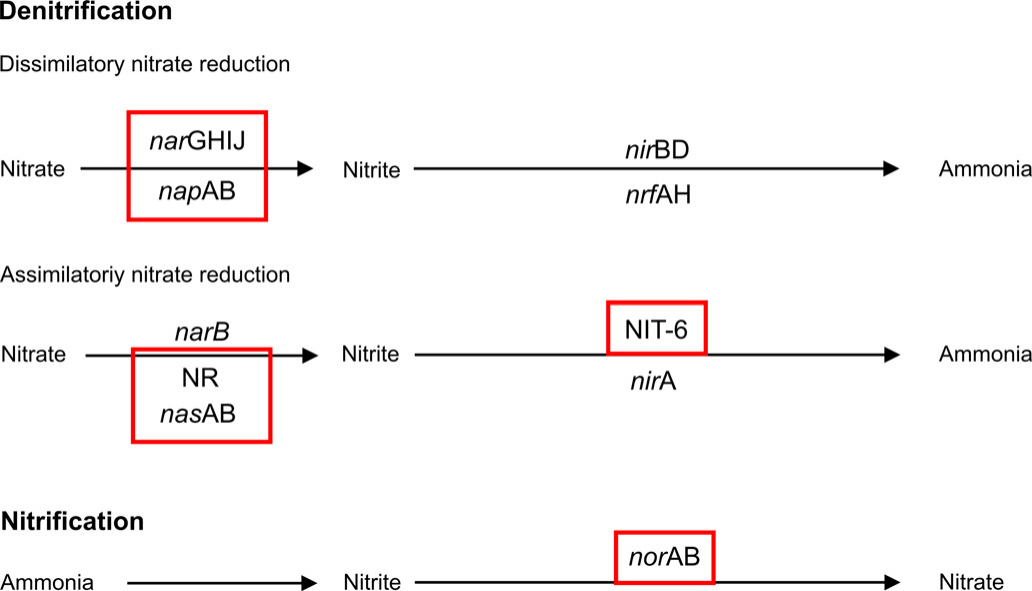
Nitrogen metabolism analysis. Simplified reaction steps for dissimilatory and assimilatory denitrification and nitrification with the corresponding genes encoding the reactions. Depicted in red rectangles are predicted genes with significant higher abundances at active community level in the fertilizer-treated soils.

To assess the functional impact of fertilizer application functional profiles were calculated from 16S rRNA data. Principal component analysis (PCA) of these profiles revealed differences between entire and active bacterial communities (S5 Fig). Moreover, significant difference between the soil communities in fertilized and non-fertilized plots were not recorded (S5 Fig). Further analysis revealed that the abundance of 1,420 genes was significantly different with respect to fertilizer application at DNA level. 54% of these were significantly more abundant in the fertilized plots and 46% in non-fertilized plots. At RNA level, the abundance of 1,012 genes was significantly increased or decreased in response to fertilizer application. 750 of these genes were significantly more abundant in fertilized plots and 262 in non-fertilized plots. Higher abundances of genes encoding for subunits of nitrate reductases (*nar*IJ) and nitrite reductases (*nir*B) were recorded in fertilized plots at active bacterial community level (Fig 8). Moreover, genes facilitating the first step of the nitrification reaction (*amo*ABC) were more abundant in fertilized soils. In summary, fertilizer application increased nitrate/nitrite uptake, denitrification, and nitrification steps in the bacterial community composition. Thus, fertilizer application enhanced most of the nitrogen-related metabolism except nitrogen fixation.

## Conclusion

Due to the importance of soil bacterial communities for ecosystem functioning, it is of importance to analyze the main drivers of these communities. In this study, the active and entire bacterial communities in fertilized and non-fertilized grassland soils were investigated over two constitutive years. In agreement with our first hypothesis, we showed that fertilizer application altered the structure and the diversity of the entire and active bacterial community. This alteration was stronger at active bacterial community level, and led to a diversity loss and shift to taxonomic groups able to use N compounds for respiratory processes. In accordance with our first and third hypotheses, fertilizer amendment increased the abundance of phylogenetic groups performing nitrate/nitrite uptake, denitrification, and nitrification steps. In addition, a higher abundance of the corresponding genes was recorded at active bacterial community level. In contrast to our second hypothesis, we showed that sampling year impacts bacterial diversity, but only at active bacterial community level. Sampling time affected only a few phyla and orders and changes were stronger in the active bacterial community. In addition, the results indicated the presence of a stable core community, which is able to adapt to environmental changes. Correlation analyses of soil properties and the relative abundances of bacterial phyla and orders suggest that soil pH and C/N ratio were good predictors for bacterial community composition and diversity. The analysis revealed stronger correlations of the active bacterial community in fertilized than in non-fertilized soils. The observed changes in dynamics and functions of bacterial soil communities as response to season and fertilizer application might contribute to a better understanding of ecosystem services provided by soil bacteria.

## Supporting Information

S1 Fig Rarefaction curves at 20% genetic distance calculated for the entire bacterial community in fertilized plots (fe_DNA:20%), active bacterial community in fertilized plots (fe_RNA:20%), entire bacterial community in non-fertilized plots (nf_DNA:20%), and active bacterial community in non-fertilized plots (nf_RNA:20%). (PDF)

S2 Fig Rarefaction curves at 3% genetic distance calculated for the entire bacterial community in fertilized plots (fe_DNA:3%), active bacterial community in fertilized plots (fe_RNA:3%), entire bacterial community in non-fertilized plots (nf_DNA:3%), and active bacterial community in non-fertilized plots (nf_RNA:3%). (PDF)

S3 Fig Relative abundances of rare phyla (< 1% abundance) derived from the analyzed soil samples. Fertilized (fe) and non-fertilized (nf) samples are shown in this figure. Samples were taken in April (Apr), July (Jul), and September (Sep) in 2010 (10) and 2011 (11) and the entire (D) and active (R) bacterial communities were analyzed.
(PDF)

S4 Fig Boxplot diagram of the number of taxonomic units (OTUs) at 20% genetic distance over sampling time at DNA and RNA level. **1**; April 2010, **2**; July 2010, **3**; September 2010, **4**; April 2011, **5**; July 2011, and **6**; September 2011.
(PDF)

S5 Fig Redundancy analysis (RDA) of the functional bacterial community profiles derived from fertilizer and non-fertilizer treatments (A), and entire and active bacterial communities (B). (PDF)

S1 Table Climatic conditions in 2010 and 2011 at the sampling site. (PDF)

S2 Table Soil properties in fertilized and non-fertilized samples (PDF)

S3 Table Number of 16S rRNA gene sequences derived from the analyzed soil samples. (PDF)

S4 Table Chao1, Michaelis-Menten-Fit (MMF), observed OTUs, Shannon indices, Simpson indices and coverage at 20% genetic distance (phylum level) calculated for fertilized soil samples. (PDF)

S5 Table Chao1, Michaelis-Menten-Fit (MMF), observed OTUs, Shannon indices, Simpson indices and coverage at 20% genetic distance (phylum level) calculated for non-fertilized soil samples. (PDF)

S6 Table Chao1, Michaelis-Menten-Fit (MMF), observed OTUs, Shannon indices, Simpson indices and coverage at 3% genetic distance (species level) calculated for fertilized soil samples. (PDF)

S7 Table Chao1, Michaelis-Menten-Fit (MMF), observed OTUs, Shannon indices, Simpson indices and coverage at 3% genetic distance (species level) calculated for non-fertilized soil samples. (PDF)
