# Supplementary material for: Effects of Fertilization and Sampling Time on Composition and Diversity of Entire and Active Bacterial Communities in German Grassland Soils
Source: PLoS One. 2015 Dec 22;10(12):e0145575. doi: 10.1371/journal.pone.0145575 (PMC4687936; doi:10.1371/journal.pone.0145575)
Supplement: S1 Fig — (PDF) [file pone.0145575.s001.pdf]

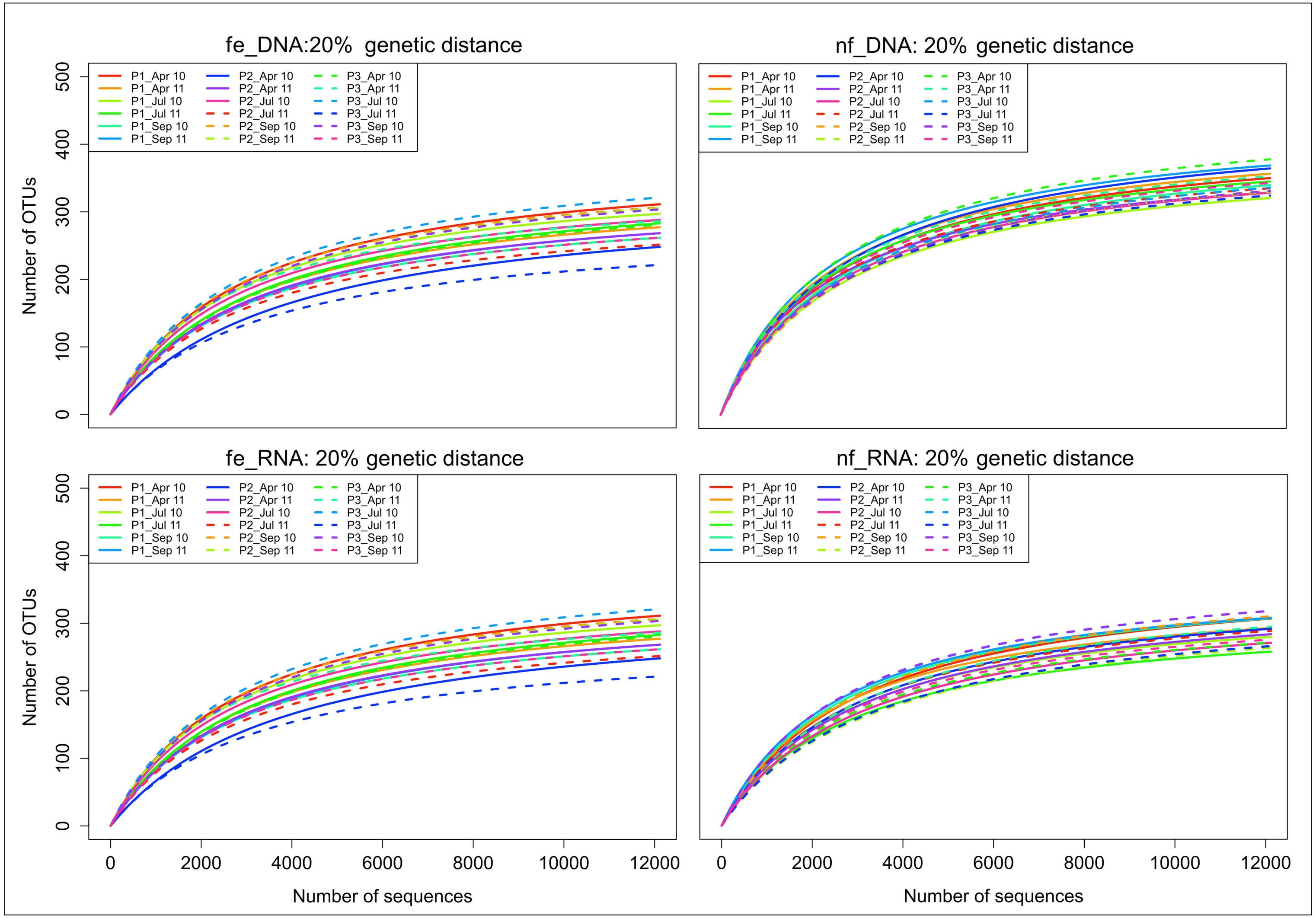

Figure S1. Rarefaction curves at 20% genetic distance calculated for the entire bacterial community in fertilized plots (fe_DNA:20%), active bacterial community in fertilized plots (fe_RNA:20%), entire bacterial community in non-fertilized plots (nf_DNA:20%), and active bacterial community in non-fertilized plots (nf_RNA:20%).
