# Supplementary material for: Effects of Fertilization and Sampling Time on Composition and Diversity of Entire and Active Bacterial Communities in German Grassland Soils
Source: PLoS One. 2015 Dec 22;10(12):e0145575. doi: 10.1371/journal.pone.0145575 (PMC4687936; doi:10.1371/journal.pone.0145575)
Supplement: S3 Fig — Fertilized (fe) and non-fertilized (nf) samples are shown in this figure. Samples were taken in April (Apr), July (Jul), and September (Sep) in 2010 (10) and 2011 (11) and the entire (D) and active (R) bacterial communities were analyzed. (PDF) [file pone.0145575.s003.pdf]

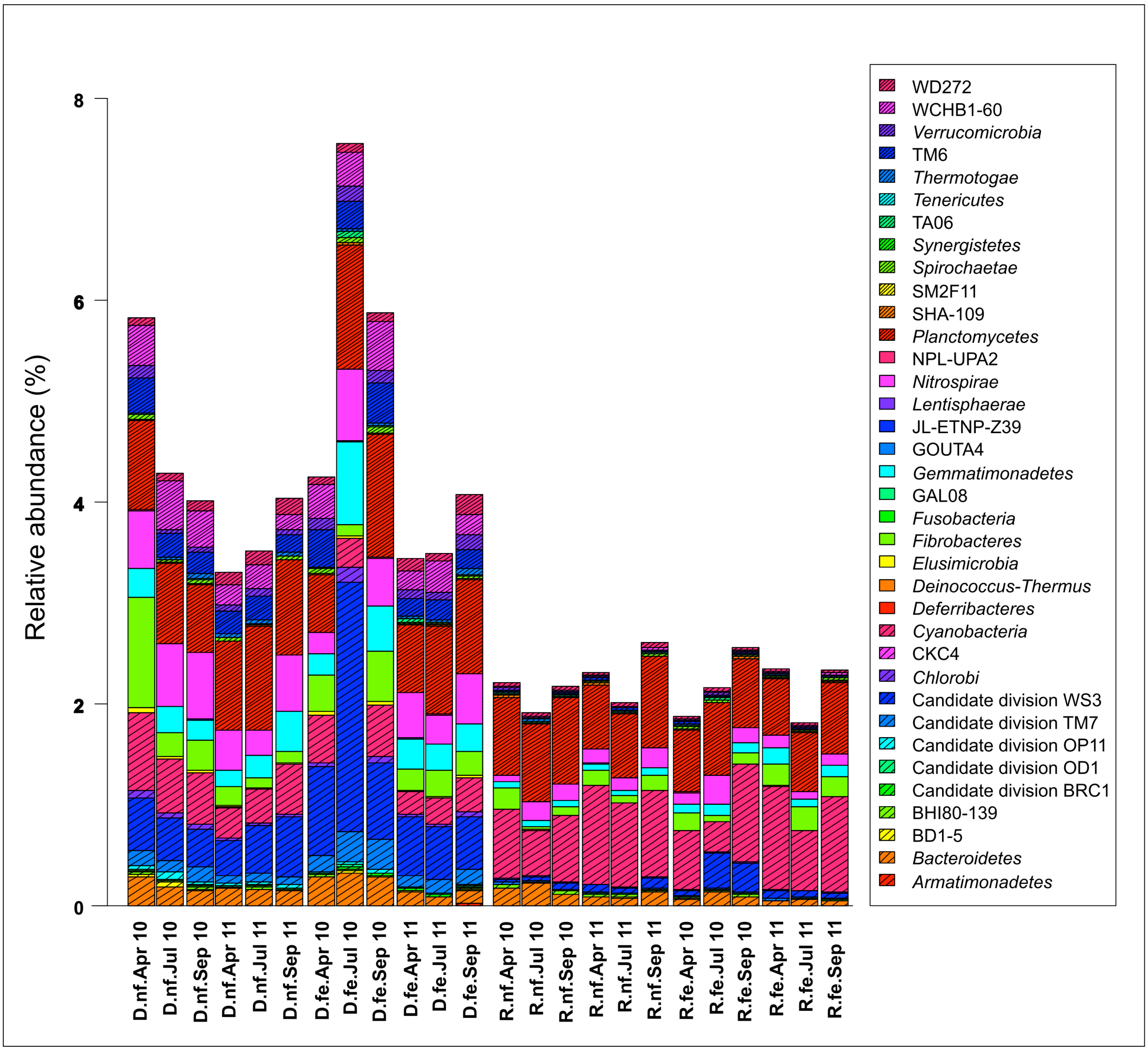

Figure S3: Relative abundances of rare phyla (< 1% abundance) derived from the analyzed soil samples. Fertilized (fe) and non-fertilized (nf) samples are shown in this figure. Samples were taken in April (Apr), July (Jul), and September (Sep) in 2010 (10) and 2011 (11) and the total (D) and active (R) bacterial communities were analyzed.
