# Supplementary material for: Effects of Fertilization and Sampling Time on Composition and Diversity of Entire and Active Bacterial Communities in German Grassland Soils
Source: PLoS One. 2015 Dec 22;10(12):e0145575. doi: 10.1371/journal.pone.0145575 (PMC4687936; doi:10.1371/journal.pone.0145575)
Supplement: S4 Fig — 1; April 2010, 2; July 2010, 3; September 2010, 4; April 2011, 5; July 2011, and 6; September 2011. (PDF) [file pone.0145575.s004.pdf]

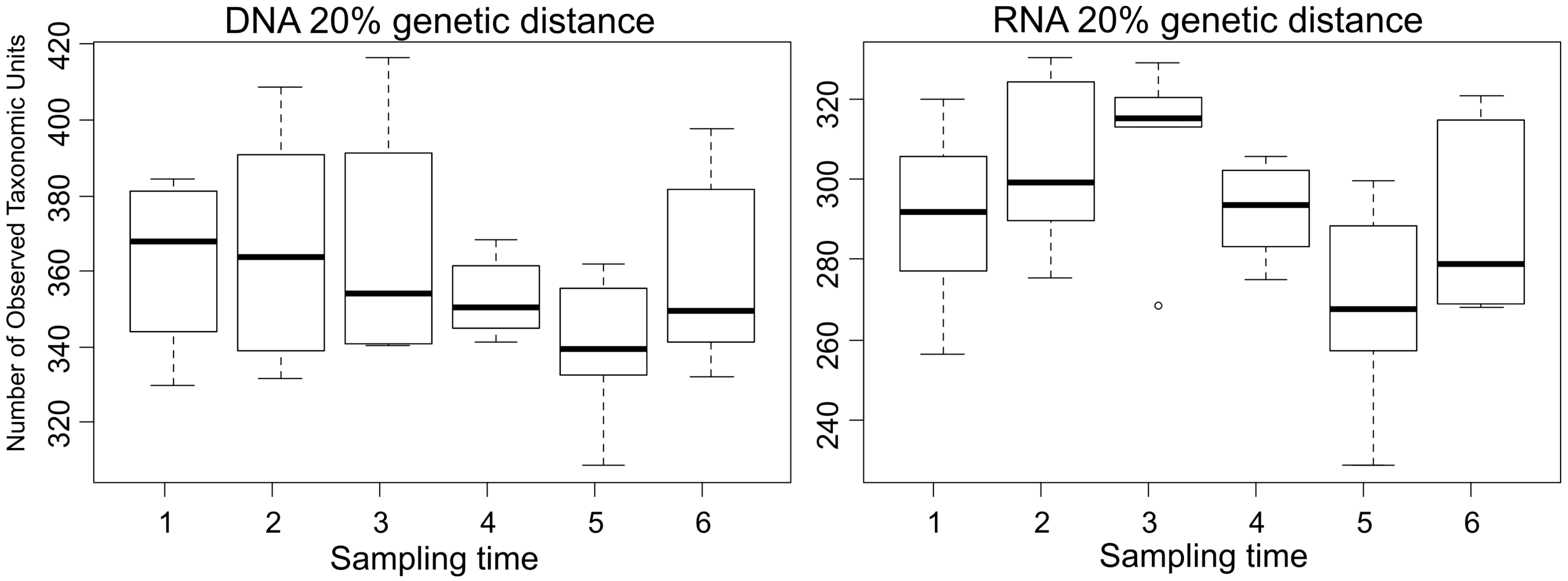

Figure S4. Boxplot diagram of the number of taxonomic units (OTUs) at 20% genetic distance over sampling time at DNA and RNA level. 1; April 2010, **2**; July 2010, **3**; September 2010, **4**; April 2011, **5**; July 2011, and **6**; September 2011.
