# Supplementary material for: Effects of Fertilization and Sampling Time on Composition and Diversity of Entire and Active Bacterial Communities in German Grassland Soils
Source: PLoS One. 2015 Dec 22;10(12):e0145575. doi: 10.1371/journal.pone.0145575 (PMC4687936; doi:10.1371/journal.pone.0145575)
Supplement: S5 Fig — (PDF) [file pone.0145575.s005.pdf]

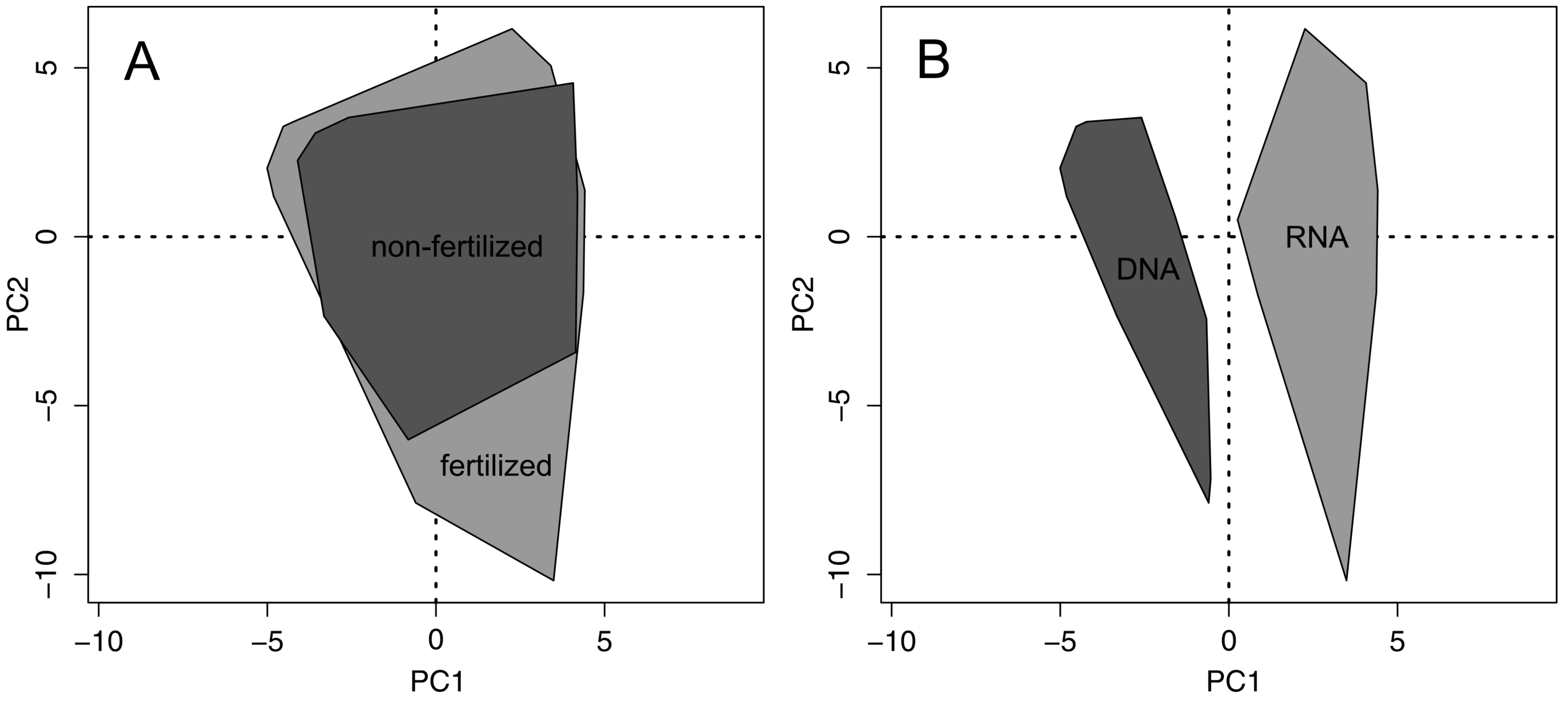

Figure S5. Redundancy analysis (RDA) of the functional bacterial community profiles derived from fertilizer and non-fertilizer treatments (A), and entire and active bacterial communities (B).
