# Supplementary material for: Effects of Fertilization and Sampling Time on Composition and Diversity of Entire and Active Bacterial Communities in German Grassland Soils
Source: PLoS One. 2015 Dec 22;10(12):e0145575. doi: 10.1371/journal.pone.0145575 (PMC4687936; doi:10.1371/journal.pone.0145575)
Supplement: S1 Table — (PDF) [file pone.0145575.s006.pdf]

Table S1. Climatic conditions in 2010 and 2011 at the sampling site. Sampling times in 2010 and 2011 are indicated by bold letters.

Month	Mean temperature (°C)		Mean precipitation (mm)	
	2010	2011	2010	2011
January	-4.72	0.15	n.d.	19.4
February	-2.62	0.05	n.d.	35.5
March	1.93	4.42	53.13	6
**April**	**7.78**	**11.26**	**14.84**	**41.75**
May	8.45	12.71	113.39	23.25
June	15.65	15.17	26.45	60.5
**July**	**19.87**	**14.48**	**47.27**	**110.85**
August	15.36	16.62	181.70	125.25
**September**	**11.42**	**14.75**	**102.12**	**54.75**
October	7.65	9.24	37.835	69.5
November	3.24	5.52	155.135	15.25
December	-4.81	2.51	n.d.	162
