# Supplementary material for: Effects of Fertilization and Sampling Time on Composition and Diversity of Entire and Active Bacterial Communities in German Grassland Soils
Source: PLoS One. 2015 Dec 22;10(12):e0145575. doi: 10.1371/journal.pone.0145575 (PMC4687936; doi:10.1371/journal.pone.0145575)
Supplement: S2 Table — (PDF) [file pone.0145575.s007.pdf]

Table S2. Soil properties in fertilized and non-fertilized samples.

**Sample/ plot**	**Sampling time**	**Plot treatment**	**pH value (+KCl) ± SD**	**Water content ± SD (%)**	**C/N**
fe1.apr10	spring 2010	fertilized	4.9±0.2	27.8±2.3	14.2
fe2.apr10	spring 2010	fertilized	4.8±0.1	27.6±1.9	12.3
fe3.apr10	spring 2010	fertilized	4.2±0.4	28.9±1.4	13.3
fe1.jul10	summer2010	fertilized	5.4±0.6	16.3±4.7	13.5
fe2.jul10	summer 2010	fertilized	4.6±0.2	13.1±1.2	12.0
fe3.jul10	summer 2010	fertilized	4.3±0.2	13.4±1.0	12.3
fe1.sep10	autumn 2010	fertilized	4.6±0.9	24.9±0.7	14.5
fe2.sep10	autumn 2010	fertilized	4.5±0.0	23.9±1.9	13.6
fe3.sep10	autumn 2010	fertilized	4.6±0.2	24.9±0.7	13.0
nf1.apr10	spring 2010	non-fertilized	4.8±0.1	28.4±1.5	13.3
nf2.apr10	spring 2010	non-fertilized	4.8±0.2	28.2±0.6	15.2
nf3.apr10	spring 2010	non-fertilized	4.6±0.1	28.1±0.8	14.6
nf1.jul10	summer2010	non-fertilized	4.9±0.2	12.6±1.1	11.8
nf2.jul10	summer 2010	non-fertilized	4.5±0.1	13.6±0.5	11.1
nf3.jul10	summer 2010	non-fertilized	4.9±0.2	13.6±3.4	11.9
nf1.sep10	autumn 2010	non-fertilized	4.7±0.2	24.4±0.8	13.1
nf2.sep10	autumn 2010	non-fertilized	4.8±0.3	25.1±2.6	13.8
nf3.sep10	autumn 2010	non-fertilized	4.5±0.1	23.9±1.8	13.1
fe1.apr11	spring 2011	fertilized	6.2±1.2	25.2±1.1	13.3
fe2.apr11	spring 2011	fertilized	4.6±0.1	25.6±1.4	12.7
fe3.apr11	spring 2011	fertilized	4.7±0.1	24.6±1.1	13.7
fe1.jul11	summer2011	fertilized	4.8±0.0	26.5±0.1	13.8
fe2.jul11	summer 2011	fertilized	4.9±0.0	25.5±0.3	11.7
fe3.jul11	summer 2011	fertilized	4.5±0.0	24.9±0.4	13.4
fe1.sep11	autumn 2011	fertilized	6.1±1.2	33.0±1.1	14.5
fe2.sep11	autumn 2011	fertilized	4.4±0.0	33.8±1.6	12.5
fe3.sep11	autumn 2011	fertilized	4.5±0.1	33.2±0.7	12.9
nf1.apr11	spring 2011	non-fertilized	4.7±0.2	23.8±0.7	14.1
nf2.apr11	spring 2011	non-fertilized	4.5±0.2	24.7±0.4	13.3
nf3.apr11	spring 2011	non-fertilized	4.3±0.1	25.0±1.1	13.9
nf1.jul11	summer2011	non-fertilized	4.7±0.0	24.5±0.6	12.6
nf2.jul11	summer 2011	non-fertilized	4.7±0.0	23.8±0.8	13.4
nf3.jul11	summer 2011	non-fertilized	4.6±0.0	25.5±0.4	14.7
nf1.sep11	autumn 2011	non-fertilized	4.9±0.2	31.9±1.2	12.7
nf2.sep11	autumn 2011	non-fertilized	4.8±0.1	34.0±0.8	11.7
nf3.sep11	autumn 2011	non-fertilized	4.6±0.2	32.7±2.1	14.7
