# Supplementary material for: Effects of Fertilization and Sampling Time on Composition and Diversity of Entire and Active Bacterial Communities in German Grassland Soils
Source: PLoS One. 2015 Dec 22;10(12):e0145575. doi: 10.1371/journal.pone.0145575 (PMC4687936; doi:10.1371/journal.pone.0145575)
Supplement: S3 Table — (PDF) [file pone.0145575.s008.pdf]

Table S3. Number of 16S rRNA gene sequences derived from the analyzed soil samples

Sample/plot	Season	Year	Treatment	Type	No. of sequences ≥ 200 bp
fe.1.apr10.D	April	2010	fertilized	DNA	33037
fe.2.apr10.D	April	2010	fertilized	DNA	51918
fe.3.apr10.D	April	2010	fertilized	DNA	63036
fe.1.jul10.D	July	2010	fertilized	DNA	42250
fe.2.jul10.D	July	2010	fertilized	DNA	23727
fe.3.jul10.D	July	2010	fertilized	DNA	20785
fe.1.sep10.D	September	2010	fertilized	DNA	68334
fe.2.sep10.D	September	2010	fertilized	DNA	32079
fe.3.sep10.D	September	2010	fertilized	DNA	22040
fe.1.apr11.D	April	2011	fertilized	DNA	11804
fe.2.apr11.D	April	2011	fertilized	DNA	26113
fe.3.apr11.D	April	2011	fertilized	DNA	26294
fe.1.jul11.D	July	2011	fertilized	DNA	24935
fe.2.jul11.D	July	2011	fertilized	DNA	29163
fe.3.jul11.D	July	2011	fertilized	DNA	27321
fe.1.sep11.D	September	2011	fertilized	DNA	16582
fe.2.sep11.D	September	2011	fertilized	DNA	25557
fe.3.sep11.D	September	2011	fertilized	DNA	23785
fe.1.apr10.R	April	2010	fertilized	RNA	23464
fe.2.apr10.R	April	2010	fertilized	RNA	39332
fe.3.apr10.R	April	2010	fertilized	RNA	47063
fe.1.jul10.R	July	2010	fertilized	RNA	30060
fe.2.jul10.R	July	2010	fertilized	RNA	38149
fe.3.jul10.R	July	2010	fertilized	RNA	34804
fe.1.sep10.R	September	2010	fertilized	RNA	28644
fe.2.sep10.R	September	2010	fertilized	RNA	47588
fe.3.sep10.R	September	2010	fertilized	RNA	33842
fe.1.apr11.R	April	2011	fertilized	RNA	28592
fe.2.apr11.R	April	2011	fertilized	RNA	21720
fe.3.apr11.R	April	2011	fertilized	RNA	34974
fe.1.jul11.R	July	2011	fertilized	RNA	28413
fe.2.jul11.R	July	2011	fertilized	RNA	19965
fe.3.jul11.R	July	2011	fertilized	RNA	32437
fe.1.sep11.R	September	2011	fertilized	RNA	29549
fe.2.sep11.R	September	2011	fertilized	RNA	26764
fe.3.sep11.R	September	2011	fertilized	RNA	41785

Table S2 (continued).

Sample/plot	Season	Year	Treatment	Type	No. of sequences ≥ 200 bp
nf.1.apr10.D	April	2010	non-fertilized	DNA	57027
nf.2.apr10.D	April	2010	non-fertilized	DNA	38753
nf.3.apr10.D	April	2010	non-fertilized	DNA	22641
nf.1.jul10.D	July	2010	non-fertilized	DNA	48278
nf.2.jul10.D	July	2010	non-fertilized	DNA	41926
nf.3.jul10.D	July	2010	non-fertilized	DNA	37297
nf.1.sep10.D	September	2010	non-fertilized	DNA	34497
nf.2.sep10.D	September	2010	non-fertilized	DNA	72754
nf.3.sep10.D	September	2010	non-fertilized	DNA	31675
nf.1.apr11.D	April	2011	non-fertilized	DNA	19889
nf.2.apr11.D	April	2011	non-fertilized	DNA	24163
nf.3.apr11.D	April	2011	non-fertilized	DNA	20192
nf.1.jul11.D	July	2011	non-fertilized	DNA	28212
nf.2.jul11.D	July	2011	non-fertilized	DNA	32871
nf.3.jul11.D	July	2011	non-fertilized	DNA	20910
nf.1.sep11.D	September	2011	non-fertilized	DNA	21413
nf.2.sep11.D	September	2011	non-fertilized	DNA	30533
nf.3.sep11.D	September	2011	non-fertilized	DNA	18547
nf.1.apr10.R	April	2010	non-fertilized	RNA	42641
nf.2.apr10.R	April	2010	non-fertilized	RNA	38801
nf.3.apr10.R	April	2010	non-fertilized	RNA	48222
nf.1.jul10.R	July	2010	non-fertilized	RNA	27221
nf.2.jul10.R	July	2010	non-fertilized	RNA	53503
nf.3.jul10.R	July	2010	non-fertilized	RNA	23597
nf.1.sep10.R	September	2010	non-fertilized	RNA	28239
nf.2.sep10.R	September	2010	non-fertilized	RNA	72380
nf.3.sep10.R	September	2010	non-fertilized	RNA	25334
nf.1.apr11.R	April	2011	non-fertilized	RNA	27139
nf.2.apr11.R	April	2011	non-fertilized	RNA	28004
nf.3.apr11.R	April	2011	non-fertilized	RNA	17919
nf.1.jul11.R	July	2011	non-fertilized	RNA	28802
nf.2.jul11.R	July	2011	non-fertilized	RNA	36424
nf.3.jul11.R	July	2011	non-fertilized	RNA	32771
nf.1.sep11.R	September	2011	non-fertilized	RNA	36845
nf.2.sep11.R	September	2011	non-fertilized	RNA	22847
nf.3.sep11.R	September	2011	non-fertilized	RNA	38062
