# Supplementary material for: Effects of Fertilization and Sampling Time on Composition and Diversity of Entire and Active Bacterial Communities in German Grassland Soils
Source: PLoS One. 2015 Dec 22;10(12):e0145575. doi: 10.1371/journal.pone.0145575 (PMC4687936; doi:10.1371/journal.pone.0145575)
Supplement: S4 Table — (PDF) [file pone.0145575.s009.pdf]

Table S4. Chao1, Michaelis-Menten-Fit (MMF), observed OTUs, Shannon indices, Simpson indices and coverage at 20% genetic distance (phylum level) calculated for fertilized soil samples

Sample	Obs. OTUs	MMF	Coverage MMF (%)	Chao1	Coverage Chao1 (%)	Shannon index	Simpson index
fe.1.apr10.D	329.90	393.10	84	437.507	75	3.43	0.911
fe.1.apr10.R	319.80	382.31	84	430.709	74	2.94	0.84
fe.1.apr11.D	345.10	408.86	84	444.849	78	3.39	0.889
fe.1.apr11.R	283.30	341.05	83	386.429	73	2.76	0.825
fe.1.jul10.D	380.70	438.87	87	483.727	79	4.04	0.958
fe.1.jul10.R	305.90	361.79	85	413.385	74	2.85	0.827
fe.1.jul11.D	361.90	430.12	84	473.574	76	3.36	0.867
fe.1.jul11.R	288.20	350.55	82	381.538	76	2.65	0.804
fe.1.sep10.D	360.70	414.97	87	452.882	80	3.91	0.953
fe.1.sep10.R	268.40	324.02	83	358.117	75	2.72	0.821
fe.1.sep11.D	346.90	412.49	84	447.326	78	3.25	0.845
fe.1.sep11.R	273.50	330.65	83	371.841	74	2.56	0.789
fe.2.apr10.D	344.00	406.41	85	458.145	75	3.49	0.911
fe.2.apr10.R	256.60	329.38	78	360.876	71	2.03	0.659
fe.2.apr11.D	346.10	410.10	84	448.556	77	3.28	0.871
fe.2.apr11.R	274.90	334.73	82	366.959	75	2.48	0.762
fe.2.jul10.D	390.90	454.21	86	513.136	76	3.93	0.951
fe.2.jul10.R	292.50	349.67	84	384.654	76	2.68	0.794
fe.2.jul11.D	335.80	390.81	86	421.219	80	3.45	0.899
fe.2.jul11.R	257.50	309.40	83	360.998	71	2.51	0.779
fe.2.sep10.D	416.40	480.95	87	542.397	77	4.20	0.965
fe.2.sep10.R	314.40	370.67	85	403.574	78	2.97	0.84
fe.2.sep11.D	397.60	457.80	87	510.454	78	3.99	0.954
fe.2.sep11.R	314.60	379.36	83	414.781	76	2.82	0.808
fe.3.apr10.D	381.40	451.12	85	504.026	76	3.41	0.866
fe.3.apr10.R	287.80	353.93	81	390.033	74	2.48	0.761
fe.3.apr11.D	354.90	418.62	85	457.392	78	3.37	0.874
fe.3.apr11.R	294.60	342.84	86	382.745	77	2.93	0.837
fe.3.jul10.D	408.60	473.83	86	542.977	75	4.09	0.961
fe.3.jul10.R	330.10	394.74	84	438.216	75	2.99	0.841
fe.3.jul11.D	308.60	365.04	85	404.501	76	3.05	0.832
fe.3.jul11.R	228.70	283.85	81	321.617	71	2.16	0.732
fe.3.sep10.D	391.50	449.44	87	494.759	79	3.99	0.95
fe.3.sep10.R	313.10	376.32	83	410.953	76	2.94	0.846
fe.3.sep11.D	332.30	389.26	85	445.925	75	3.35	0.88
fe.3.sep11.R	268.00	323.90	83	359.501	75	2.53	0.79
