# Supplementary material for: Effects of Fertilization and Sampling Time on Composition and Diversity of Entire and Active Bacterial Communities in German Grassland Soils
Source: PLoS One. 2015 Dec 22;10(12):e0145575. doi: 10.1371/journal.pone.0145575 (PMC4687936; doi:10.1371/journal.pone.0145575)
Supplement: S5 Table — (PDF) [file pone.0145575.s010.pdf]

Table S5. Chao1, Michaelis-Menten-Fit (MMF), observed OTUs, Shannon indices, Simpson indices and coverage at 20% genetic distance (phylum level) calculated for non-fertilized soil samples.

Sample	Obs. OTUs	MMF	Coverage MMF (%)	Chao1	Coverage Chao1 (%)	Shannon index	Simpson index
nf.1.apr10.D	360.00	422.16	85	477.4	75	3.49	0.902
nf.1.apr10.R	305.70	368.37	83	415.49	74	2.83	0.83
nf.1.apr11.D	368.20	430.12	86	482.098	76	3.25	0.823
nf.1.apr11.R	305.50	364.59	84	406.166	75	3.01	0.855
nf.1.jul10.D	331.60	392.58	84	437.343	76	3.48	0.916
nf.1.jul10.R	289.50	346.89	83	395.266	73	2.70	0.81
nf.1.jul11.D	355.70	414.37	86	458.683	78	3.63	0.918
nf.1.jul11.R	263.50	316.36	83	363.799	72	2.49	0.758
nf.1.sep10.D	348.00	411.87	84	474.055	73	3.57	0.921
nf.1.sep10.R	315.60	379.17	83	414.391	76	2.93	0.842
nf.1.sep11.D	381.60	444.95	86	486.216	78	3.77	0.933
nf.1.sep11.R	320.60	373.80	86	438.322	73	3.29	0.889
nf.2.apr10.D	375.80	447.55	84	488.227	77	3.53	0.903
nf.2.apr10.R	295.90	355.86	83	389.304	76	2.79	0.829
nf.2.apr11.D	341.50	396.03	86	442.91	77	3.51	0.917
nf.2.apr11.R	292.70	351.37	83	421.47	69	2.67	0.806
nf.2.jul10.D	339.10	401.16	85	464.493	73	3.56	0.931
nf.2.jul10.R	275.50	337.29	82	367.932	75	2.50	0.765
nf.2.jul11.D	342.90	399.02	86	438.666	78	3.52	0.915
nf.2.jul11.R	299.50	362.24	83	405.644	74	2.54	0.777
nf.2.sep10.D	340.40	407.87	83	439.648	77	3.39	0.891
nf.2.sep10.R	320.20	386.88	83	429.841	74	2.87	0.827
nf.2.sep11.D	341.50	404.73	84	448.923	76	3.26	0.875
nf.2.sep11.R	268.90	334.93	80	368.097	73	2.40	0.765
nf.3.apr10.D	384.20	451.95	85	492.195	78	3.79	0.943
nf.3.apr10.R	277.10	335.78	83	365.574	76	2.62	0.802
nf.3.apr11.D	361.60	422.34	86	468.792	77	3.52	0.899
nf.3.apr11.R	302.10	366.67	82	408.509	74	2.58	0.782
nf.3.jul10.D	346.80	413.84	84	467.899	74	3.64	0.941
nf.3.jul10.R	324.10	389.60	83	419.702	77	2.99	0.85
nf.3.jul11.D	332.50	391.18	85	454.457	73	3.29	0.874
nf.3.jul11.R	271.80	335.95	81	379.09	72	2.41	0.756
nf.3.sep10.D	340.80	406.60	84	455.856	75	3.66	0.945
nf.3.sep10.R	329.10	395.05	83	430.621	76	2.88	0.804
nf.3.sep11.D	352.20	414.40	85	447.24	79	3.50	0.904
nf.3.sep11.R	283.90	339.19	84	383.635	74	2.67	0.809
