# Supplementary material for: Effects of Fertilization and Sampling Time on Composition and Diversity of Entire and Active Bacterial Communities in German Grassland Soils
Source: PLoS One. 2015 Dec 22;10(12):e0145575. doi: 10.1371/journal.pone.0145575 (PMC4687936; doi:10.1371/journal.pone.0145575)
Supplement: S7 Table — (PDF) [file pone.0145575.s012.pdf]

Table S7. Chao1, Michaelis-Menten-Fit (MMF), observed OTUs, Shannon indices, Simpson indices and coverage at 3% genetic distance (species level) calculated for non-fertilized soil samples

Sample	Obs. OTUs	MMF	Coverage MMF (%)	Chao1	Coverage Chao1 (%)	Shannon index	Simpson index
nf.1.apr10.D	2,880.50	6,374.64	45	7,631.01	38	5.98	0.97
nf.1.apr10.R	2,435.70	5,128.78	47	6,267.89	39	5.54	0.96
nf.1.apr11.D	3,600.00	8,344.67	43	9,629.26	37	6.59	0.98
nf.1.apr11.R	2,419.70	5,353.16	45	6,388.14	38	5.42	0.96
nf.1.jul10.D	2,938.80	6,409.38	46	7,744.69	38	6.08	0.98
nf.1.jul10.R	3,259.20	7,750.36	42	8,626.49	38	6.18	0.98
nf.1.jul11.D	3,376.30	7,529.48	45	8,930.88	38	6.51	0.98
nf.1.jul11.R	2,394.10	5,908.84	41	6,950.88	34	4.90	0.92
nf.1.sep10.D	3,032.90	6,858.83	44	8,108.52	37	5.93	0.96
nf.1.sep10.R	3,112.20	7,287.10	43	8,723.44	36	6.03	0.97
nf.1.sep11.D	3,239.90	7,151.90	45	8,733.32	37	6.39	0.98
nf.1.sep11.R	2,401.40	5,400.19	44	6,368.17	38	5.28	0.95
nf.2.apr10.D	2,950.20	6,044.58	49	7,178.80	41	6.43	0.99
nf.2.apr10.R	2,731.70	5,895.18	46	7,040.79	39	5.90	0.97
nf.2.apr11.D	3,402.70	8,023.85	42	9,263.07	37	6.33	0.97
nf.2.apr11.R	2,643.30	6,209.03	43	7,535.76	35	5.46	0.96
nf.2.jul10.D	2,521.40	4,686.95	54	5,794.76	44	6.33	0.99
nf.2.jul10.R	2,413.70	5,337.76	45	6,506.61	37	5.38	0.96
nf.2.jul11.D	3,006.40	6,854.37	44	8,309.71	36	6.10	0.97
nf.2.jul11.R	2,256.50	5,149.60	44	6,264.86	36	5.10	0.95
nf.2.sep10.D	2,636.20	4,989.75	53	6,535.58	40	6.24	0.98
nf.2.sep10.R	3,167.20	7,413.04	43	8,623.58	37	6.08	0.97
nf.2.sep11.D	3,078.60	6,902.05	45	8,050.91	38	6.11	0.97
nf.2.sep11.R	2,325.70	5,727.57	41	6,665.32	35	4.83	0.92
nf.3.apr10.D	3,891.40	8,703.98	45	0,062.35	39	7.01	0.99
nf.3.apr10.R	2,952.20	6,921.75	43	8,134.66	36	5.84	0.97
nf.3.apr11.D	3,023.90	7,490.27	40	8,839.03	34	5.69	0.95
nf.3.apr11.R	2,823.80	6,707.73	42	7,384.02	38	5.59	0.96
nf.3.jul10.D	2,506.20	4,947.26	51	6,186.17	41	5.92	0.98
nf.3.jul10.R	3,306.70	8,004.72	41	8,706.97	38	6.08	0.97
nf.3.jul11.D	3,272.80	7,811.78	42	8,807.20	37	6.22	0.98
nf.3.jul11.R	2,817.20	6,887.81	41	7,675.72	37	5.50	0.95
nf.3.sep10.D	2,630.50	5,442.65	48	6,742.54	39	6.07	0.99
nf.3.sep10.R	3,162.80	7,590.92	42	8,698.21	36	5.91	0.96
nf.3.sep11.D	2,956.30	6,610.75	45	7,512.26	39	6.03	0.98
nf.3.sep11.R	2,722.00	5,999.56	45	6,976.53	39	5.72	0.97
